# ﻿New insights into the phylogeny of Tetrigoidea (Insecta, Orthoptera), with the announcement of the first mitogenome of the genus *Phaesticus*

**DOI:** 10.3897/zookeys.1251.154178

**Published:** 2025-09-04

**Authors:** Xuejuan Li, Xiaomei Dong, Liliang Lin

**Affiliations:** 1 College of Life Sciences, Shaanxi Normal University, Xi’an, China Shaanxi Normal University Xi’an China; 2 Zoological and Botanical Museum, Shaanxi Normal University, Xi’an, China Shaanxi Normal University Xi’an China

**Keywords:** Caelifera, divergence time, mitogenome, *
Phaesticus
*, phylogeny, pygmy grasshoppers, Tetrigoidea

## Abstract

The mitochondrial genome (mitogenome) has been widely used to infer the phylogeny, origin and evolution of insects. Although mitogenomic data have been used to study the phylogenetic relationships of Tetrigoidea (Orthoptera), larger sample sizes were also essential to explore the detailed phylogenetic relationships of these taxa. In this study, two complete mitogenomic sequences were sequenced from *Phaesticus moniliantennatus* (formerly *Flatocerus daqingshanensis* and *F. nankunshanensis*). The mitogenome sequences were assembled, annotated and analyzed. The length of the mitogenomes was 16,147 and 16,224 bp, and the nucleotide composition was A>T>C>G, A-skew and C-skew. Large intergenic regions between trnS(ucn) and ND1 were identified with a length of 191–233 bp. Phylogenetic analyses showed that Batrachideinae formed the basal position of Tetrigoidea, followed by Tripetalocerinae. The monophyly of several subfamilies was not supported. Within Tetriginae, *P. moniliantennatus* clustered with a clade containing *Systolederus spicupennis*, *S. hainanensis* and *S. zhengi* (Metrodorinae), indicating their closer phylogenetic relationship. The divergence time results indicated that Batrachideinae diverged at 170.96 Ma and Tripetalocerinae diverged at 149.36 Ma. And the divergence time between *P. moniliantennatus* and the closely related *Systolederus* clade was 87.06 Ma. These results represent the available mitogenome sequences of the genus *Phaesticus* and provide a valuable data resource for reconstructing phylogenetic relationships and studying the differentiation of Tetrigoidea species.

## ﻿Introduction

The animal mitochondrial genome (mitogenome), with a size of ~16 kb, contains 37 genes, i.e., 13 protein-coding genes (PCGs), two ribosomal RNA genes (rRNAs) and 22 transfer RNA genes (tRNAs) (Boore 1999). Characteristics such as maternal inheritance, low recombination rate and high rate of evolution have made mitogenome sequences ideal molecular markers for studying animal phylogeny, phylogeography, origin and evolution. For example, mitogenomes have been widely used to study mitogenome sequence characteristics, gene order evolution, phylogenetic relationships, and divergence time in orthopterans (Song et al. 2015; Chang et al. 2020; Gaugel et al. 2023).

Orthoptera insects have been grouped into two clades, namely Ensifera and Caelifera (Song et al. 2015). Among Caelifera, the superfamily Tridactyloidea was at the basal position, followed by Tetrigoidea, and the divergence time between these two superfamilies was 260.44 Ma. Tetrigoidea species, the pygmy grasshoppers, are the second most species-rich superfamily within Caelifera (Song et al. 2015). They are small in size, widely distributed around the world and considered agricultural pests. The species constitute their own family (Tetrigidae) [Orthoptera Species File (OSF) v. 5.0] (Cigliano et al. 2025), which includes seven subfamilies (Batrachideinae, Cladonotinae, Lophotettiginae, Metrodorinae, Scelimeninae, Tetriginae, Tripetalocerinae) and two tribes (Criotettigini, Thoradontini). However, some studies have grouped them into nine subfamilies of Tetrigidae (Scelimeninae, Metrodorinae, Diseotettiginae, Tetriginae, Cladonotinae, Lophotettiginae, Batrachideinae, Tripetalocerinae, and Cleostratinae) (Deng 2016; Li et al. 2021a). The phylogeny of Tetrigoidea species has been studied using mitochondrial genes or whole mitogenomes (Lin et al. 2015; Lin et al. 2017; Li et al. 2021a; Qin et al. 2023; Guan et al. 2024; Luo et al. 2024), but due to limited sampling, comprehensive phylogenetic relationships require further analysis.

The species of genus *Flatocerus* were previously classified into Discotettiginae, Tetrigidae and Tetrigoidea (Zheng 2005), and most previous studies on this genus mainly focused on morphological classification (Zheng et al. 2011; Ding et al. 2017; Zha et al. 2021; Skejo et al. 2022). In terms of molecular data, a previous phylogenetic study based on the mitochondrial COI gene showed that *Flatocerus nankunshanensis* is closely related to *Systolederus spicupennis* and *S. guangxiensis* (Metrodorinae) (Lin et al. 2015). And *F. nankunshanensis* clustered with *S. guangxiensis*, and then grouped with *S. spicupennis* in maximum parsimony (MP), maximum likelihood (ML) and Bayesian inference (BI) trees, while *F. nankunshanensis* first clustered with *S. spicupennis* and then grouped with *S. guangxiensis* in the neighbor-joining (NJ) tree (Lin et al. 2015).

In contrast, the genus *Flatocerus* is considered synonymous with *Phaesticus* based on the Orthoptera Species File (v. 5.0) (Cigliano et al. 2025), and *F. daqingshanensis* and *F. nankunshanensis* are considered synonymous with *Phaesticus moniliantennatus* (Zha et al. 2021). Regarding the phylogenetic status of the genus *Phaesticus*, Skejo (2017) considered it to belong to Metrodorinae or Tetriginae, while Zha et al. (2021) proposed it as an independent branch closer to Metrodorinae and Tetriginae. The taxonomic placement of *Phaesticus* needs to be further investigated based on molecular phylogenetic evidence (Zha et al. 2021). However, to date, there are no records of complete mitogenome sequences associated with *Phaesticus* in the GenBank database, only single mitochondrial gene sequences, including the COI and CYTB genes. Single mitochondrial genes contain limited phylogenetic information, which is probably insufficient to reconstruct their evolutionary history. Therefore, complete mitogenomes of *Phaesticus* are needed to further investigate their genome characteristics and evolution.

This study is the first to sequence, assemble and annotate mitogenomes from *P. moniliantennatus* (formerly *F. daqingshanensis* and *F. nankunshanensis*). The genome sequence features were first analyzed. Then, phylogenetic relationships were reconstructed using the mitochondrial datasets in combination with other Tetrigidae mitogenomes from the GenBank database. In addition, the divergence time of Tetrigidae species was analyzed using the mitogenome datasets. These results provide newly available mitogenome resources for *Phaesticus*, a new perspective on their phylogenetic implications within Tetrigoidea, and additional clues for traditional classification.

## ﻿Material and methods

### ﻿Taxon sampling, DNA extraction and sequencing

Sample 1 of *P. moniliantennatus* (formerly *F. daqingshanensis*) was collected in 2012 in Shiwandashan, Guangxi, China (21°86.52'N, 107°88.90'E), and sample 2 of *P. moniliantennatus* (formerly *F. nankunshanensis*) was collected in 2011 in Emeishan, Sichuan, China (29°56.84'N, 103°35.09'E). The specimens were stored at -20 °C and deposited at Shaanxi Normal University, Shaanxi Province, China. Total genomic DNA was extracted using a TIANGEN DP802 kit, and the quality was checked using the Qubit Fluorometer. The DNA was fragmented using an ultrasonic-mechanical method to prepare a small-inserted-fragment library, and the data were sequenced using the Illumina X-plus platform with a paired-end read length of 150 bp.

### ﻿Mitogenome assembly, annotation and analysis

Adaptor sequences were removed from the raw data using FASTP (Chen et al. 2018a), and low-quality reads were filtered using FASTQ-FILTER. Sequences were assembled using NOVOPLASTY v. 4.3.1 (Dierckxsens et al. 2017), with the *Tetrix japonica* mitogenome sequence (NC_018543) as the seed and reference sequence. The assembled mitogenomes were first annotated using the MITOS webserver (Bernt et al. 2013) and then compared with other available Tetrigoidea mitogenome annotation information for verification, including *T. japonica* and other closely related species. Among these, the position and secondary structure of tRNAs were referenced in comparison with other Tetrigoidea mitogenomes (Lin et al. 2017). Nucleotide composition, relative synonymous codon usage (RSCU) and genetic distance were calculated in MEGA v. 11 (Tamura et al. 2021). Base skew was calculated using the formula AT-skew=[(A-T)/(A+T)] and GC-skew [(G-C)/(G+C)] (Perna and Kocher 1995). Tandem repeats were predicted using Tandem Repeat Finder with default settings (Benson 1999).

### ﻿Phylogenetic inference

A total of 41 Tetrigoidea mitogenome sequences were used for phylogenetic analyses, including six subfamilies (Batrachideinae, Tripetalocerinae, Scelimeninae, Cladonotinae, Tetriginae and Metrodorinae) and two tribes (Criotettigini and Thoradontini) in Tetrigidae, as well as two Tridactyloidea outgroups (*Ellipes minuta* and *Mirhipipteryx andensis*) (Suppl. material 1). Two mitogenome datasets (PCG and PCG+rRNA+tRNA) were used for phylogenetic analyses, with the PCG dataset containing 13 PCGs and the PCG+rRNA+tRNA dataset containing 13 PCGs, two rRNAs and 22 tRNAs. The mitogenome sequences for each gene were aligned individually using the Muscle program in MEGA v. 11 (Tamura et al. 2021), with the PCG aligned using codon-based strategies. For each PCG, stop codons were removed, amino acids aligned and then converted to the corresponding nucleotide sequences. SEQUENCEMATRIX v. 1.7.8 (Vaidya et al. 2011) was used to concatenate the mitogenome datasets.

Phylogenetic analyses were performed using ML and BI methods. The best-fitting model was estimated using the MODELFINDER program in PHYLOSUITE v. 1.2.3 (Zhang et al. 2020) under the Bayesian information criterion (BIC). The GTR+F+I+I+R4 model was used to reconstruct the ML tree for each dataset, while the GTR+F+I+G4 model was used for the BI tree. ML analyses were performed in IQ-TREE v. 2.1.3 (Nguyen et al. 2015) with 1000 bootstrap replicates. BI analyses were performed in MRBAYES v. 3.2.7 (Ronquist et al. 2012), with two independent runs of four simultaneous Markov chains. Ten million generations were run, sampling every 100 trees. The first 25% of trees were discarded as burn-in, and the remaining trees were used to obtain the consensus tree. Effective sample size (ESS) values were estimated in TRACER v. 1.5 (Rambaut et al. 2004) to ensure that the ESS value was greater than 200.

### ﻿Divergence time

The divergence time of the sampled Tetrigoidea species was estimated using the MCMCTREE program in PAML v. 4.9 (Yang 1997). The PCG dataset and corresponding ML tree were used, and four records from the TimeTree website (Kumar et al. 2017) were used as calibration points, including 4.4–8.0 Ma between *T. japonica* and *Alulatettix yunnanensis*, 135 Ma between *T. japonica* and *Trachytettix bufo*, 131.2–271.1 Ma between *T. japonica* and *E. minuta*, 153.5–186.0 Ma between *E. minuta* and *M. andensis*. The JC69 model was used, with other parameters set to burn-in of 100,000, sampfreq of 50 and nsample of 500,000.

## ﻿Results and discussion

### ﻿Mitogenome structure and organization

Reads of 24,062,441 and 24,098,467 after trimming were used for the assembly of *P. moniliantennatus*. Two newly sequenced mitogenome sequences of *P. moniliantennatus* were obtained, and the mitogenomes were submitted to the GenBank database with accession numbers PQ767100 and PQ767101. The length of the whole mitogenome sequences was 16,147 bp and 16,224 bp. The nucleotide composition was similar, with 43.3% A, 16.3% C, 9.8% G and 30.6% T in *P. moniliantennatus* (PQ767101) and 43.3% A, 15.6% C, 10% G and 31.2% T in PQ767100. The A+T content (73.9% and 74.5%) was significantly higher than the G+C content (Suppl. material 2: fig. S1a). A- and C-skew were found throughout the mitogenomes (Suppl. material 2: fig. S1b, c).

**Figure 1.**
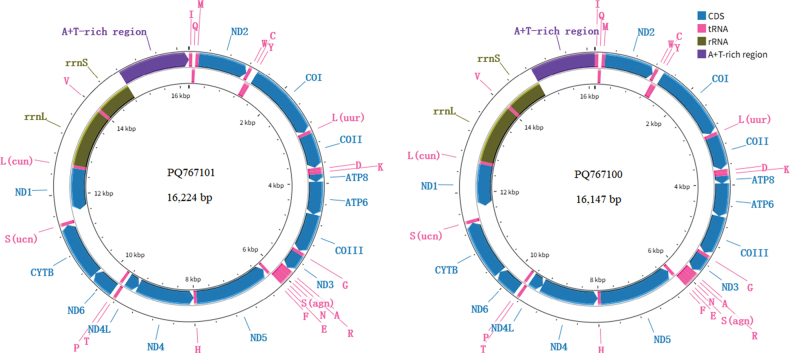
Mitogenome organization of *Phaesticus moniliantennatus*.

The gene composition and structure of the *P. moniliantennatus* mitogenomes are shown in Fig. 1, which contains 37 mitochondrial genes (13 protein-coding genes (PCGs), two RNAs, 22 tRNAs) and one non-coding region (A+T-rich region). The mitogenome organization, gene order, and coding strand were consistent with those of other Tetrigoidea species (Li et al. 2021a, b; Qin et al. 2023; Guan et al. 2024; Luo et al. 2024).

An intergenic region of 191 bp was identified between trnS(ucn) and ND1 in the mitogenome of *P. moniliantennatus* (PQ767101), while 233 bp was identified in the mitogenome of PQ767100. These larger intergenic regions between trnS(ucn) and ND1 have also been found in other Tetrigoidea mitogenomes, with some species containing tandem repeat sequences in this region (Li et al. 2021a). However, no tandem repetitive sequences were identified in these relatively large intergenic regions of *P. moniliantennatus*.

### ﻿Protein-coding genes

Nine PCGs were encoded on the J-strand, and the remaining four PCGs (ND5, ND4, ND4L and ND1) were encoded on the N-strand (Fig. 1). The total PCGs contained showed T- and C-skew tendencies (Suppl. material 2: fig. S1b, c), which is consistent with other Tetrigoidea mitogenomes (Li et al. 2021a). Among the different PCG datasets, the A+T content was higher than that of G+C, especially in the PCG-3^rd^ dataset (Suppl. material 2: fig. S1a). An obvious T-skew was found in the PCG-2^nd^ and PCG-N datasets (Suppl. material 2: fig. S1b). An obvious G-skew was found in the PCG-1^st^ and PCG-N datasets, while an obvious C-skew was identified in the PCG-J dataset (Suppl. material 2: fig. S1c).

Four initiation codons were identified in *P. moniliantennatus* (PQ767101) PCGs, including ATA (ND2, COII, ND3, ND1), ATT (ATP8, COIII), ATC (CYTB), ATG of the remaining six PCGs, while three termination codons were found in PCGs, including TAG (ND3, ND4, CYTB, ND1), incomplete stop codon T (COI, COIII, ND5), TAA of the remaining six PCGs. In addition, four pattern initiation codons were used in *P. moniliantennatus* (PQ767100) PCGs, including ATT (ND2, ND4L), ATC (COI), ATA (ND3, ND1), and ATG in the remaining eight PCGs, while three termination codons were found in PCGs, including TAG (ND4, CYTB), incomplete T (COI, COIII, ND3, ND5), and TAA of the remaining seven PCGs. The incomplete T can generate functional termination codons via post-transcriptional polyadenylation (Ojala et al. 1981).

The RSCU results showed that the codon variation tendency was similar between the two *P. moniliantennatus* individuals. The codons with the third position ending in A and U were most frequently used (Suppl. material 3), which is consistent with other Tetrigoidea mitogenomes (Li et al. 2021a). The five most frequently used codons were UUA(L), UCU(S), CGA(R), UCA(S) and ACA(T) in that order in *P. moniliantennatus* (PQ767101) and UUA(L), UCU(S), UCA(S), ACA(T) and GUU(V) in that order in PQ767100.

The p-distance result based on the COI gene showed that the p-value ranged from 0.0026 (between *Thoradonta nodulosa* and *T. obtusilobata*) to 0.2740 (between *Tripetaloceroides tonkinensis* and *Bolivaritettix yuanbaoshanensis*) (Suppl. material 4), with an average value of 0.2058. Between two individuals of the same species, the p-value was 0.0026 for *T. japonica* and 0.1475 for *P. moniliantennatus*. In addition, Chen et al. (2018b) calculated the K2P-distance distance of 24 Scelimeninae species (representing nine genera) based on the combined sequences of COI, rrnL and 18S rRNA gene. The results showed that the average value within Scelimeninae species was 0.126, with the highest value (0.169) found between *Scelimena melli* and *Zhengitettix curvispinus*.

### ﻿RNA genes

Two rRNAs (rrnL and rrnS) were encoded on the N-strand. The rrnL gene was located between trnL(cun) and trnV, and rrnS was located between trnV and the A+T-rich region (Fig. 1). The length of the rrnL genes was 1310 bp in *P. moniliantennatus* (PQ767101) and 1314 bp in PQ767100, and the length of rrnS was 789 bp in *P. moniliantennatus* (PQ767101) and 800 bp in PQ767100. The rRNAs also showed a higher A+T content than that of G+C (Suppl. material 2: fig. S1a). Obvious T- and G-skew were found in the rRNAs (Suppl. material 2: fig. S1b, c).

There were 14 tRNAs encoded on the J-strand, while the remaining eight tRNAs (trnQ, trnC, trnY, trnF, trnH, trnP, trnL(cun) and trnV) were encoded on the N-strand (Fig. 1). The total length of the tRNAs was 1454 bp in *P. moniliantennatus* (PQ767101) and 1458 bp in PQ767100. The tRNA datasets showed a higher content of A+T than G+C (Suppl. material 2: fig. S1a), and an obvious G-skew was found in the tRNA-N dataset (Suppl. material 2: fig. S1c).

The length of 22 tRNAs ranged from 60 bp (trnR) to 72 bp (trnV) in *P. moniliantennatus* (PQ767101) and from 60 bp (trnR) to 74 bp (trnV) in PQ767100. All tRNAs formed a typical clover secondary structure with four stems: acceptor (AA) arm, dihydrouridine (DHU) arm, anticodon (AC) arm and TψC arm, except for trnS(agn), which lacked the DHU arm (Suppl. material 5). This structure of the trnS(agn) gene was a common feature and was also found in other Caelifera species (Zhang et al. 2013; Wang et al. 2023). The nucleotide composition and secondary structure of tRNAs were conserved between two individuals of *P. moniliantennatus*. For example, several stem and loop structures of tRNAs were consistent, such as the DHU arm and the loop of trnI (Suppl. material 5).

### ﻿A+T-rich region

The non-coding region of the A+T-rich region was located between rrnS and trnI (Fig. 1), with a length of 1412 bp in *P. moniliantennatus* (PQ767101) and 1270 bp in PQ767100. A significantly higher A+T content was found in the A+T-rich region (Suppl. material 2: fig. S1a), with 84.6% in *P. moniliantennatus* (PQ767101) and 85.7% in PQ767100. A- and C-skew were identified in this region (Suppl. material 2: fig. S1b, c). Tandem repetitive sequences of 214 bp were identified in *P. moniliantennatus* (PQ767101) with a copy number 4.0 (Suppl. material 6: fig. S5a), while two types of tandem repetitive sequences were identified in PQ767100, including 214 bp (copy number 1.8) and 57 bp (copy number 2.2) (Suppl. material 6: fig. S5b).

### ﻿Phylogenetic reconstruction

**Figure 2.**
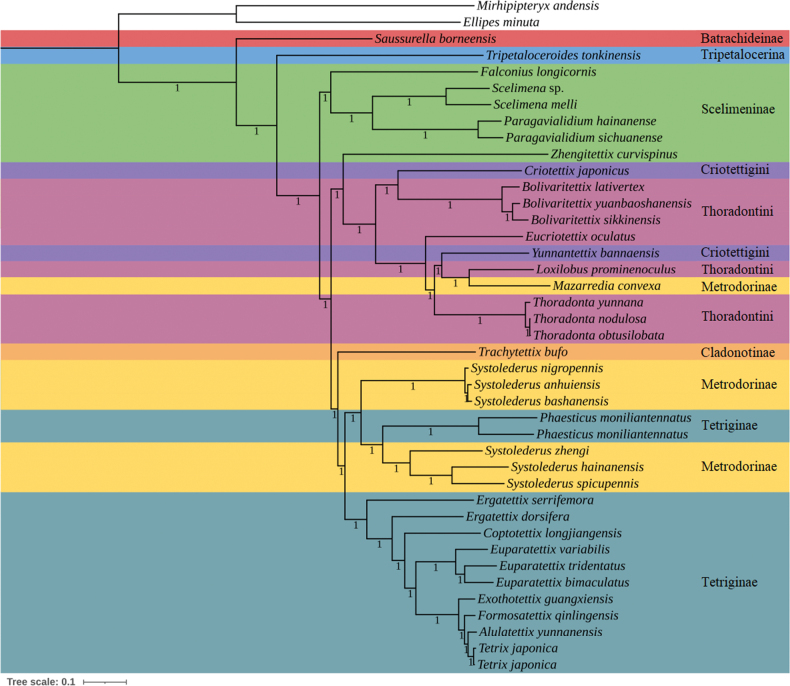
Phylogenetic tree reconstructed from the PCG+rRNA+tRNA dataset using BI method.

Phylogenetic reconstruction revealed a consistent topology (Fig. 2, Suppl. material 7), with relatively higher bootstrap values (BSs) in ML trees and posterior probabilities (PPs) in BI trees. Species from six subfamilies (Batrachideinae, Tripetalocerinae, Scelimeninae, Metrodorinae, Cladonotinae and Tetriginae) and two tribes (Criotettigini and Thoradontini) were included in this study, but none were monophyletic except for Batrachideinae, Tripetalocerinae and Cladonotinae (only one species within each group). The non-monophyly of several Tetrigoidea groups has been noted in previous studies (Li et al. 2021a; Qin et al. 2023; Luo et al. 2024). For example, Adžić et al. (2020) proposed that Scelimeninae is a polyphyletic group composed of genera that contain species with spiky lateral lobes, and the shape of the lateral lobes of the paranota does not seem to be a good character to distinguish subfamilies. Additionally, Scelimeninae was also non-monophyletic in our reconstructed phylogenetic trees.

Batrachideinae occupied the basal position of Tetrigoidea, with PP=1 between *Saussurella borneensis* and the remaining species, a phylogeny consistent with previous studies (Luo et al. 2024). Tripetalocerinae diverged from the phylogeny, with BS=100 and PP=1 between *Tripetaloceroides tonkinensis* and other Tetrigoidea species (Fig. 2 and Suppl. material 7), which is also consistent with previous studies (Luo et al. 2024).

*Zhengitettix curvispinus* formed the basal position of the clade containing *Criotettix japonicus*/*Bolivaritettix*/*Eucriotettix oculatus*/*Yunnantettix bannaensis*/*Loxilobus prominenoculus*/*Mazarredia convexa*/*Thoradonta* in our study, whereas *Z. curvispinus* occupied a relatively basal position among Tetrigoidea species, with the exception of Batrachideinae and Tripetalocerinae (Luo et al. 2024). This result suggested that *Zhengitettix* species may contain relatively greater differences compared to other Scelimeninae species, and extensive sampling of the genus *Zhengitettix* is needed to further explore its phylogeny. In addition, several previous studies have found that *Z. curvispinus* is not clustered with other Scelimeninae members (Li et al. 2021a, b; Luo et al. 2024). These findings suggest that the genus *Zhengitettix* does not belong to Scelimeninae, a conclusion requiring validation with a larger sample collection.

According to the OSF v. 5.0, the genus *Yunnantettix* belongs to Cladonotinae, whereas the genus *Bolivaritettix* is best assigned to Metrodorinae. However, both genera were consistently grouped within Criotettigini/Thoradontini, consistent with previous studies (Li et al. 2021a; Luo et al. 2024).

The monophyly of the genus *Systolederus* has been recovered in previous studies (Guan et al. 2024; Luo et al. 2024), but in our study, *Systolederus* was embedded in the Tetriginae species, with *P. moniliantennatus*, *S. zhengi*, *S. hainanensis* and *S. spicupennis* forming one clade, and *S. nigropennis*, *T. anhuiensis* and *S. bashanensis* forming another. These results suggest that the genus *Systolederus* has a closer phylogenetic relationship with the *Phaesticus*. A previous study showed that *F. nankunshanensis* clustered with *P. mellerborgi*, and this clade was subsequently clustered with *Bannatettix ruiliensis* (Du 2024). The clade, ((*F. nankunshanensis*, *P. mellerborgi*), *B. ruiliensis*), had a closer phylogenetic relationship with *Systolederus* species (Du 2024), consistent with the results of our phylogenetic tree. Additionally, we identified several common morphological traits between *Systolederus* and *Phaesticus*, including small body size, a narrow vertex, a long conical protuberance, oval- shaped tegmina, and well-developed hind wings. Furthermore, the phylogenetic results also suggested that the genus *Systolederus* may be polyphyletic and requires further integrative taxonomic review. The phylogenetic relationship of the genus *Systolederus* varied when different samples were used. *Systolederus* had a closer relationship with *P. moniliantennatus* in our study, but this genus formed a sister group with the genus *Macromotettixoides* (Luo et al. 2024) and occupied a basal position within Tetriginae(Guan et al. 2024). Therefore, the sampling strategy of Tetrigoidea had an important influence on their phylogenetic status.

Two specimens of *P. moniliantennatus*, representing the former *F. daqingshanensis* and *F. nankunshanensis*, respectively, were included in our study. *Flatocerus daqingshanensis* and *F. nankunshanensis* were considered synonymous with *P. moniliantennatus* by Zha et al. (2021). However, in Zha’s study, they listed several similar morphological characters, but did not provide any other evidence to support their synonymy as one species. In a previous study, *F. nankunshanensis* unsurprisingly clustered with *P. mellerborgi* (Du 2024), suggesting a close relationship between the genera *Flatocerus* and *Phaesticus*. However, these may not effectively support the synonymy of *F. daqingshanensis* and *F. nankunshanensis*, as our p-distance result based on the COI gene showed that the p-value between *F. daqingshanensis* and *F. nankunshanensis* is 0.1475, which is slightly higher than mid-range. We suggest that further research is needed on the synonymy of these two species with *P. moniliantennatus*, as there are indeed significant morphological differences, such as body shape and shape of the pronotum.

*Phaesticus moniliantennatus* was at the basal position of the clade containing *S. zhengi*, *S. hainanensis* and *S. spicupennis*, forming a phylogeny of (((*S. spicupennis*, *S. hainanensis*), *S. zhengi*), *P. moniliantennatus*), which differed from that of Lin et al. (2015). Zha et al. (2021) proposed the genus *Phaesticus* as an independent branch close to Metrodorinae and Tetriginae, but our results suggest a relatively closer phylogenetic relationship between *P. moniliantennatus* and the genus *Systolederus* (Metrodorinae) compared to other Tetriginae species.

In summary, the monophyly of several subfamilies was not recovered, suggesting that some taxonomic characters, such as the shape of the lateral lobes of the pronotum, may not be sufficient to classify Tetrigoidea species as taxonomic characters at the subfamily level. Therefore, for further study, a larger sampling of Tetrigoidea species and more molecular markers, such as UCEs (ultraconserved elements), transcriptomes, RRGS (reduced representation genome sequencing) and genome data, may help reconstruct solid phylogenetic relationships. Integrative taxonomic characters may resolve the inconsistency between morphological and molecular classification.

### ﻿Divergence time

**Figure 3.**
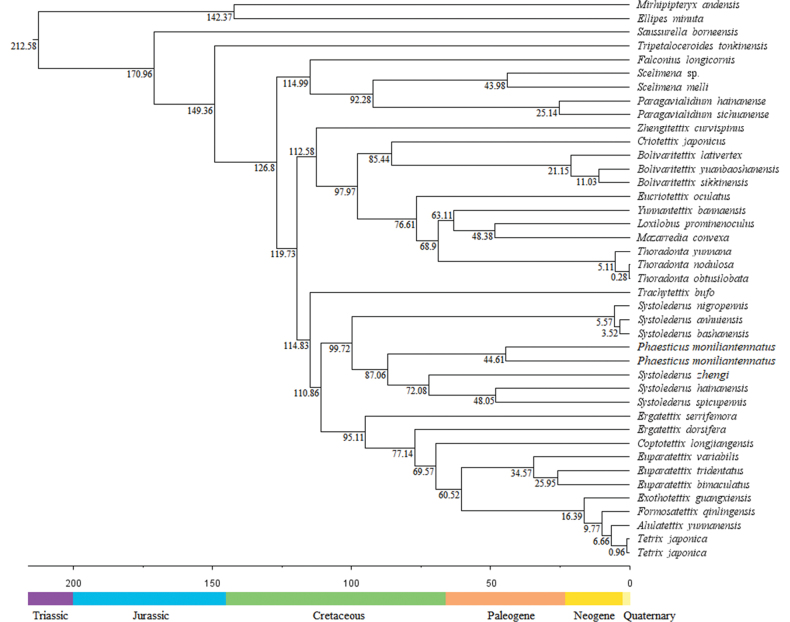
Divergence time result based on the PCG dataset.

Based on the divergence time tree, Tetrigoidea split with Tridactyloidea at 212.58 Ma during the Triassic (Fig. 3), indicating that Tetrigoidea species were one of the more ancient lineages (Song et al. 2015). In Tetrigoidea, some divergence time results were similar to previous studies, such as 0.96 Ma between *T. japonica* and *T. ruyuanensis*, compared to 0.81 Ma (Guan et al. 2024). The divergence times of different genera were relatively longer, ranging from 170.96 Ma (*Saussurella*) to 6.66 Ma (*Tetrix*), and most genera diverged during the Cretaceous. The origin and divergence of most Tetrigoidea species occurred before the Quaternary glaciation, and the glaciation may have influenced the global distribution of these species. For the basal position of Tetrigoidea, Batrachideinae, *S. borneensis* diverged at 170.96 Ma during the Jurassic, followed by *T. tonkinensis* (Tripetalocerinae) with a divergence time of 149.36 Ma also during the Jurassic (Fig. 3). For the genus *Phaesticus* within Tetriginae, the divergence time between this genus and the clade containing *S. spicupennis*, *S. hainanensis* and *S. zhengi* was 87.06 Ma during the Cretaceous. However, since only two individuals of *P. moniliantennatus* were included in the current analysis, the divergence times for this genus can be more precisely determined by adding samples of *P. mellerborgi* and *P. hainanensis* in the future.

## ﻿Conclusion

Two new mitogenome sequences of *P. moniliantennatus* (Tetrigoidea, Tetriginae) were sequenced, assembled and annotated. The mitogenome characteristics, phylogeny and divergence time were analyzed. Results based on mitogenome sequences showed that nucleotide composition, gene order, RSCU and secondary structure were similar between these two mitogenomes. The phylogenetic and divergence analyses indicated that *P. moniliantennatus* had a closer relationship with the clade of *S. spicupennis*/*S. hainanensis*/*S. zhengi* (Metrodorinae), and the split time between these two clades was 87.06 Ma.

## ﻿Additional information

### Use of AI

No use of AI was reported.

### Author ORCIDs

Xuejuan Li ￼ https://orcid.org/0000-0003-4086-6063

Xiaomei Dong ￼ https://orcid.org/0009-0001-6486-281X

Liliang Lin ￼ https://orcid.org/0000-0002-9972-4732

### Data availability

All of the data that support the findings of this study are available in the main text or Supplementary Information.

## Supplementary materials

Supplementary material 1 The mitogenome sequence data used for the phylogenetic reconstruction analysis Data type docx https://binary.pensoft.net/file/1409134This dataset is made available under the Open Database License (http://opendatacommons.org/licenses/odbl/1.0/). The Open Database License (ODbL) is a license agreement intended to allow users to freely share, modify, and use this Dataset while maintaining this same freedom for others, provided that the original source and author(s) are credited.Xuejuan Li, Xiaomei Dong, Liliang Lin

Supplementary material 2 Nucleotide composition and base skew of *Phaesticus moniliantennatus* Data type png Explanation note a: A+T content, b: AT-skew, c: GC-skew. https://binary.pensoft.net/file/1409135This dataset is made available under the Open Database License (http://opendatacommons.org/licenses/odbl/1.0/). The Open Database License (ODbL) is a license agreement intended to allow users to freely share, modify, and use this Dataset while maintaining this same freedom for others, provided that the original source and author(s) are credited.Xuejuan Li, Xiaomei Dong, Liliang Lin

Supplementary material 3 RSCU component of *Phaesticus moniliantennatus* Data type png Explanation note a: PQ767101, b: PQ767100. https://binary.pensoft.net/file/1409136This dataset is made available under the Open Database License (http://opendatacommons.org/licenses/odbl/1.0/). The Open Database License (ODbL) is a license agreement intended to allow users to freely share, modify, and use this Dataset while maintaining this same freedom for others, provided that the original source and author(s) are credited.Xuejuan Li, Xiaomei Dong, Liliang Lin

Supplementary material 4 The p-distance between Tetrigoidea species based on the COI gene Data type png Explanation note The red solid circle represented the p-value between two individuals of *Phaesticus moniliantennatus*. https://binary.pensoft.net/file/1409137This dataset is made available under the Open Database License (http://opendatacommons.org/licenses/odbl/1.0/). The Open Database License (ODbL) is a license agreement intended to allow users to freely share, modify, and use this Dataset while maintaining this same freedom for others, provided that the original source and author(s) are credited.Xuejuan Li, Xiaomei Dong, Liliang Lin

Supplementary material 5 Secondary structure of tRNAs in Phaesticus moniliantennatus (PQ767101) Data type png Explanation note The differences in PQ767100 were shown in blue, with (+) indicating insertion and (-) indicating deletion. https://binary.pensoft.net/file/1409138This dataset is made available under the Open Database License (http://opendatacommons.org/licenses/odbl/1.0/). The Open Database License (ODbL) is a license agreement intended to allow users to freely share, modify, and use this Dataset while maintaining this same freedom for others, provided that the original source and author(s) are credited.Xuejuan Li, Xiaomei Dong, Liliang Lin

Supplementary material 6 Structure of the A+T-rich region of *Phaesticus moniliantennatus* Data type png Explanation note a: PQ767101, b: PQ767100. https://binary.pensoft.net/file/1409139This dataset is made available under the Open Database License (http://opendatacommons.org/licenses/odbl/1.0/). The Open Database License (ODbL) is a license agreement intended to allow users to freely share, modify, and use this Dataset while maintaining this same freedom for others, provided that the original source and author(s) are credited.Xuejuan Li, Xiaomei Dong, Liliang Lin

Supplementary material 7 Phylogenetic trees based on three mitogenome datasets Data type png Explanation note a: PCG ML tree, b: PCG BI tree, c: PCG+rRNA+tRNA ML tree. https://binary.pensoft.net/file/1409140This dataset is made available under the Open Database License (http://opendatacommons.org/licenses/odbl/1.0/). The Open Database License (ODbL) is a license agreement intended to allow users to freely share, modify, and use this Dataset while maintaining this same freedom for others, provided that the original source and author(s) are credited.Xuejuan Li, Xiaomei Dong, Liliang Lin
