# Supplementary material for: ﻿New insights into the phylogeny of Tetrigoidea (Insecta, Orthoptera), with the announcement of the first mitogenome of the genus Phaesticus
Source: Zookeys. 2025 Sep 4;1251:115–30. doi: 10.3897/zookeys.1251.154178 (PMC12426632; doi:10.3897/zookeys.1251.154178)
Supplement: Supplementary material 1 — The mitogenome sequence data used for the phylogenetic reconstruction analysis [file zookeys-1251-115_article-154178__-s001.docx]

**Table S1.** The mitogenome sequence data used for the phylogenetic reconstruction analysis.

| **Classification** | **Species** | **Species name in OSF v.5.0** | **Accession number** |
| --- | --- | --- | --- |
| Batrachideinae | *Saussurella borneensis* |  | MZ169555 |
| Cladonotinae | *Trachytettix bufo* |  | JX913766 |
| Criotettigini | *Criotettix japonicus* |  | MT162542 |
| Criotettigini | *Yunnantettix bannaensis* |  | MN083181 |
| Metrodorinae | *Mazarredia convexa* |  | MN938924 |
| Metrodorinae | *Systolederus spicupennis* |  | MN083179 |
| Metrodorinae | *Teredorus anhuiensis* | *Systolederus anhuiensis* | NC_071822 |
| Metrodorinae | *Teredorus bashanensis* | *Systolederus bashanensis* | NC_063118 |
| Metrodorinae | *Teredorus hainanensis* | *Systolederus hainanensis* | NC_063117 |
| Metrodorinae | *Teredorus nigropennis* | *Systolederus nigropennis* | MN938922 |
| Metrodorinae | *Teredorus guangxiensis* | *Systolederus zhengi* | NC_082935 |
| Scelimeninae | *Falconius longicornis* |  | MT162543 |
| Scelimeninae | *Paragavialidium hainanense* |  | NC_071831 |
| Scelimeninae | *Paragavialidium sichuanense* |  | MT162549 |
| Scelimeninae | *Scelimena melli* |  | MW722938 |
| Scelimeninae | *Scelimena* sp. |  | OR333957 |
| Scelimeninae | *Zhengitettix curvispinus* |  | MT162544 |
| Tetriginae | *Alulatettix yunnanensis* |  | NC_018542 |
| Tetriginae | *Coptotettix longjiangensis* |  | KY798413 |
| Tetriginae | *Ergatettix dorsifera* |  | NC_046540 |
| Tetriginae | *Ergatettix serrifemora* |  | MN938923 |
| Tetriginae | *Euparatettix bimaculatus* |  | NC_046541 |
| Tetriginae | *Euparatettix tridentatus* |  | NC_082933 |
| Tetriginae | *Euparatettix variabilis* |  | NC_046542 |
| Tetriginae | *Exothotettix guangxiensis* |  | NC_082934 |
| Tetriginae | *Formosatettix qinlingensis* |  | KY798412 |
| Tetriginae | *Flatocerus daqingshanensis* | *Phaesticus moniliantennatus* | PQ767101 |
| Tetriginae | *Flatocerus nankunshanensis* | *Phaesticus moniliantennatus* | PQ767100 |
| Tetriginae | *Tetrix japonica* |  | NC_018543 |
| Tetriginae | *Tetrix ruyuanensis* | *Tetrix japonica** | NC_046412 |
| Thoradontini | *Bolivaritettix lativertex* |  | MN083173 |
| Thoradontini | *Bolivaritettix sikkinensis* |  | KY123120 |
| Thoradontini | *Bolivaritettix yuanbaoshanensis* |  | KY123121 |
| Thoradontini | *Eucriotettix oculatus* |  | MN083176 |
| Thoradontini | *Loxilobus prominenoculus* |  | MT162545 |
| Thoradontini | *Thoradonta nodulosa* |  | MT162547 |
| Thoradontini | *Thoradonta obtusilobata* |  | KY798414 |
| Thoradontini | *Thoradonta yunnana* |  | NC_071832 |
| Tripetalocerinae | *Tripetaloceroides tonkinensis* |  | MW770353 |
| Outgroup(Tridactyloidea) | *Ellipes minuta* |  | NC_014488 |
| Outgroup(Tridactyloidea) | *Mirhipipteryx andensis* |  | NC_028065 |

*: Long Y, Teng C, Huang C, Zhang R, Deng W, Lin L (2023) Twenty-three new synonyms of the Eastern common groundhopper, *Tetrix japonica* (Bolívar, 1887) (Orthoptera, Tetrigidae). Zookeys 1187: 135-167. https://doi.org/10.3897/zookeys.1187.110067
