# Supplementary figures and images for: ﻿New insights into the phylogeny of Tetrigoidea (Insecta, Orthoptera), with the announcement of the first mitogenome of the genus Phaesticus
Source: Zookeys. 2025 Sep 4;1251:115–30. doi: 10.3897/zookeys.1251.154178 (PMC12426632; doi:10.3897/zookeys.1251.154178)

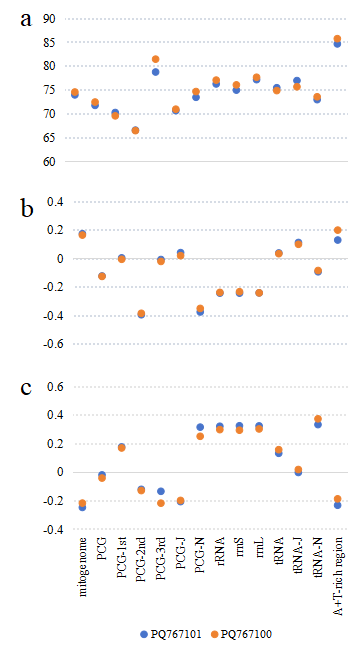

Supplement: Supplementary material 2 — Nucleotide composition and base skew of Phaesticus moniliantennatus [file zookeys-1251-115_article-154178__-s002.png]

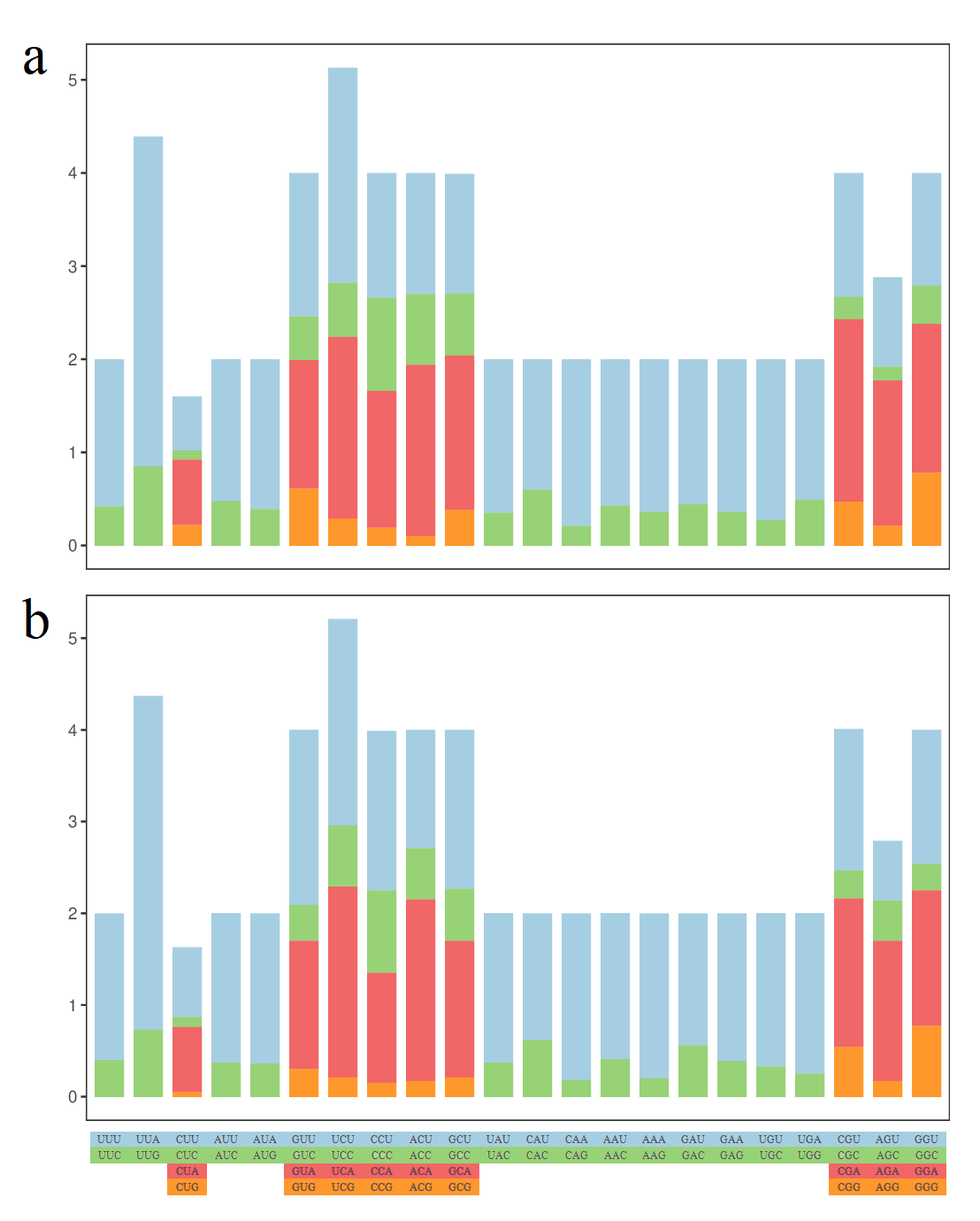

Supplement: Supplementary material 3 — RSCU component of Phaesticus moniliantennatus [file zookeys-1251-115_article-154178__-s003.png]

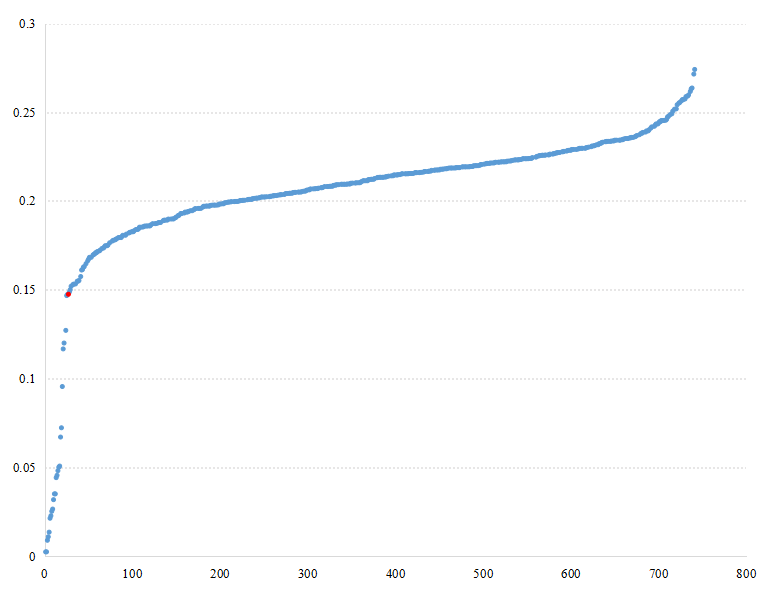

Supplement: Supplementary material 4 — The p-distance between Tetrigoidea species based on the COI gene [file zookeys-1251-115_article-154178__-s004.png]

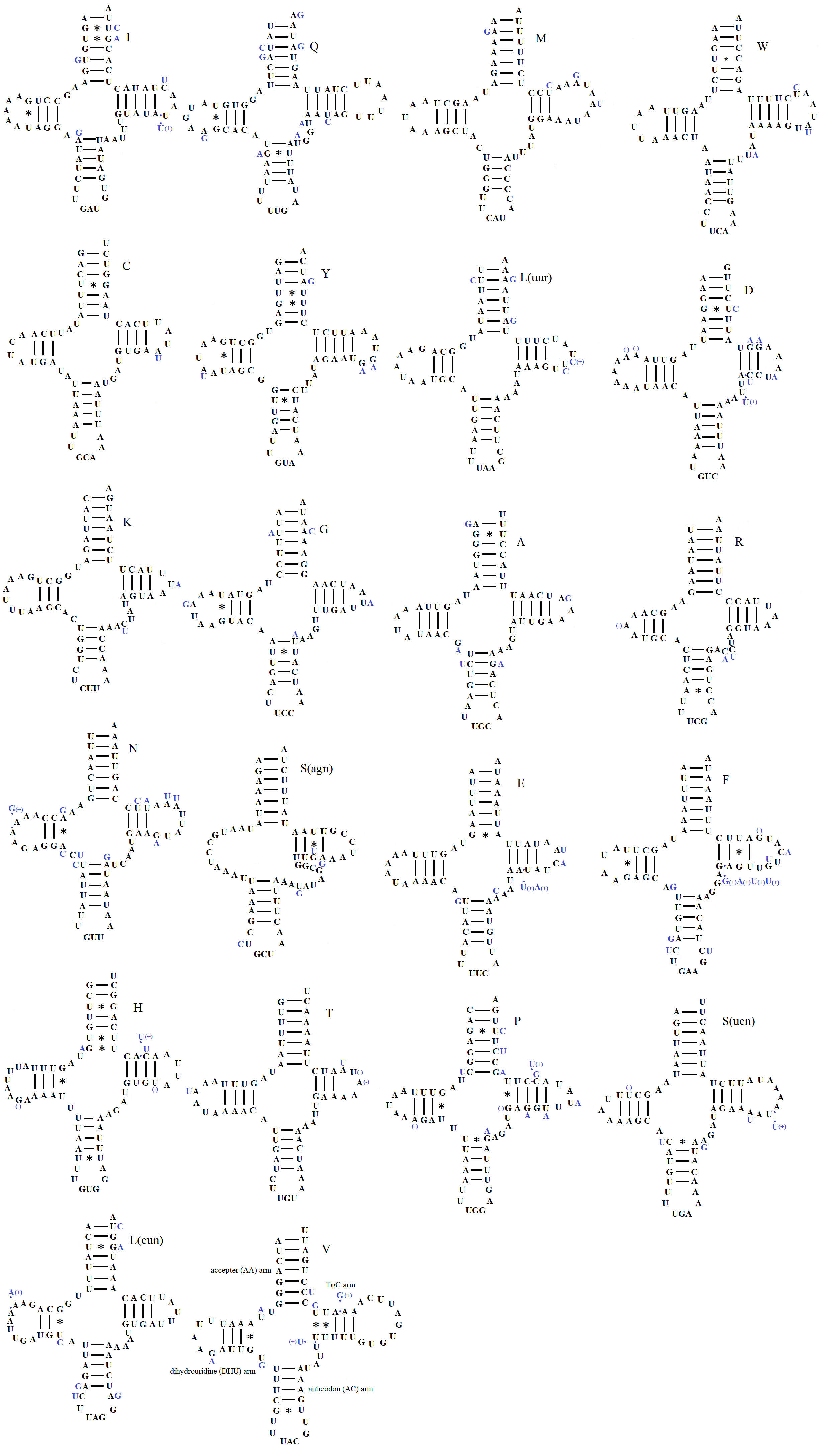

Supplement: Supplementary material 5 — Secondary structure of tRNAs in Phaesticus moniliantennatus (PQ767101) [file zookeys-1251-115_article-154178__-s005.png]

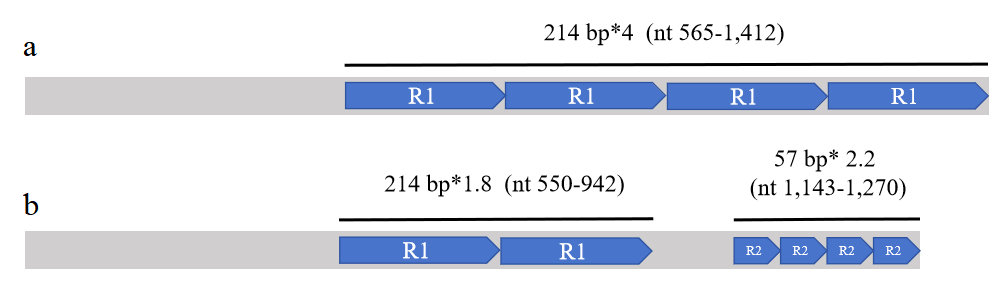

Supplement: Supplementary material 6 — Structure of the A+T-rich region of Phaesticus moniliantennatus [file zookeys-1251-115_article-154178__-s006.png]

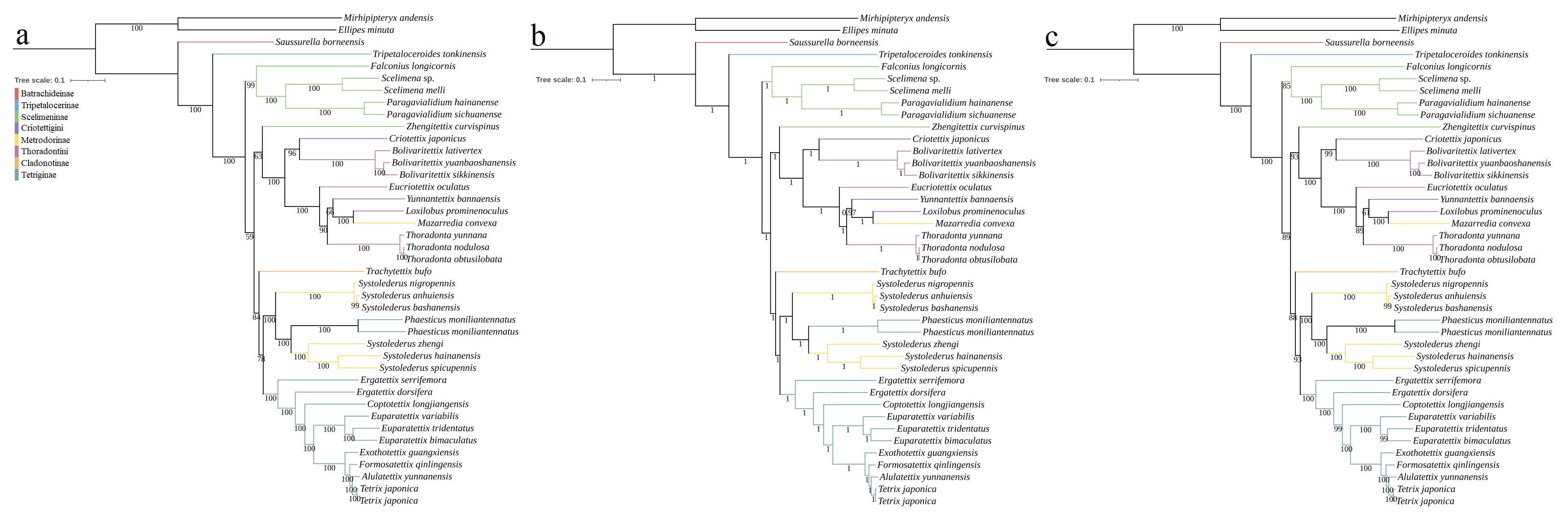

Supplement: Supplementary material 7 — Phylogenetic trees based on three mitogenome datasets [file zookeys-1251-115_article-154178__-s007.png]
